# Virtual Periacetabular Osteotomy and Anatomical Measurements

**DOI:** 10.1111/os.12438

**Published:** 2019-03-18

**Authors:** Gang Xu, Chao Dong, Krol Zdzislaw, Andreas H Krieg

**Affiliations:** ^1^ Paediatric Orthopaedic Department University Children's Hospital (UKBB) Basel Switzerland; ^2^ Department of Paediatric Orthopaedics Beijing Jishuitan Hospital Beijing China

**Keywords:** New cutting planes, Peracetabular osteotomy, Safety space, Virtual anatomical measurement

## Abstract

**Objective:**

To report on a CT scan virtual periacetabular osteotomy (PAO) process to evaluate the potential risk of different PAO cutting planes.

**Methods:**

A total of 123 patients (64 men and 59 women) underwent virtual PAO. We defined two retroacetabular cutting (RC) planes: the RC plane and the RC^+^ plane (10 mm posteriorly as compared to the RC plane). Subsequently, we measured the anatomical minimum distance between the acetabulum and the sciatic notch, the minimum distance between the acetabulum and the retroacetabular cutting plane (osteotomy of the posterior column), and the osteotomy length in the cranio‐caudal direction.

**Results:**

The mean (standard deviation [SD]) minimum distance between the acetabulum and the sciatic notch was 25.82 ± 3.52 mm (95% confidence intervals [CI], 25.36–26.25 mm). In men, the mean (SD) minimum distance between the acetabulum and sciatic notch (27.18 ± 3.47 mm; 95% CI, 26.56–27.78 mm) was significantly (3 mm) larger than in women (24.34 ± 2.92 mm; 95% CI, 23.82–24.89 mm; *P* < 0.001).

The mean (SD) minimum distance between the acetabulum and the retroacetabular plane was significantly larger for the RC^+^ plane (6.97 ± 0.91 mm) than for the RC plane (*P* < 0.001). In men, this distance (10.23 ± 3.84 mm) was significantly (2.3 mm) larger than in women (7.94 ± 3.45 mm; *P* < 0.001).

The mean (SD) osteotomy length was significantly larger for the RC^+^ plane (61.78 ± 6.75 mm) than for the RC plane (68.48 ± 6.65) mm; *P* < 0.001). All three evaluated parameters had significantly shorter lengths in women than in men.

**Conclusion:**

The safety space for PAO in women was narrower than in men. By shifting the RC plane 10 mm into the posterior direction, the RC^+^ plane provides a safer cutting distance and shorter osteotomy line in the PAO than the RC plan, which is important to avoid intraarticular penetration.

## Introduction

Due to the popularization of hip ultrasound screening and the resultant decrease in the indication for pelvic osteotomies in adolescents and young adults, the rate of operative procedures for developmental dysplasia of the hip has been reduced by approximately 50% within the past two decades^1^, ^2^. Yet, the periacetabular osteotomy (PAO) is still the most commonly used and most effective surgical procedure to treat developmental dysplasia of the hip in symptomatic adolescents and young adults^3^, ^4^. Ganz *et al.* 4 were the first to describe this widely used and effective operation method in 1988. However, PAO was also found to be associated with complication rates of 20%–25% after dorsal osteotomy. Ganz *et al.* reported that intra‐articular penetration was the most severe complication of the procedure. Other major complications of PAO include excessive bleeding and osteonecrosis of the acetabular fragment or femoral head; minor complications include iatrogenic neurovascular injury (femoral nerve or sciatic nerve palsy), deep vein thrombosis, non‐union of the osteotomy site (mainly of the superior pubic ramus), heterotopic ossification, and symptomatic screws^5^, ^6^. Different approaches, such as the modified Smith–Peterson and the ilioinguinal direct anterior approach, have been proposed to avoid these complications, none of which proved successful in this respect.

Because of the complexity of the local anatomy, PAO remains a technically demanding procedure with a distinct learning curve, especially for novices^5^, ^6^, ^7^. Ganz *et al.* 4 noted that clinically significant complications only occurred during the first 18 of 75 operations. Davey *et al.* 7 also found that major complication rates decreased from 17% to 2.9% when the first 35 cases were compared with the second 35 cases of PAO performed by one surgeon. Ferro *et al.* 8 suggested training in this technique in multiple cadavers before performing it in live patients.

Because intra‐articular penetration is a serious complication with disastrous outcomes^4^, it is important to delineate the margin of the acetabulum. To our knowledge, no reports on the key anatomic measurements of European pelvises exist to date. Such studies could clarify the margins of the hip, presumed on a quadrilateral surface, whereby PAO could be performed more safely and with reduced rates of serious complications.

By means of a virtual PAO using CT scans, PAO training could be more easily accomplished than by using cadavers. Although the cadaveric training might be more similar to a real operation, virtual PAO have the potential to reduce biases that arise from the use of different approaches, different proficiency level, different experience of the surgeons, and different learning curves, and to provide more natural and direct data from the acetabular. Therefore, the purpose of this study was: to attain useful anatomical measurements to perform virtual PAO; and to improve the intraoperative performance based on knowledge of the average size of normal pelvic structures from an adult population in Europe, as visualized by three‐dimensional (3D) CT images.

## Materials and Methods

### *Inclusion and Exclusion Criteria*

Inclusion criteria were the following: (i) patients were aged from 18 to 85 years; (ii) 3D CT images were available of bilateral views of the pelvis taken at a single hospital; (iii) availability of written informed consent; (iv) no history of hip‐related operation.

Exclusion criteria were the following: (i) history of hip‐related operations; and (ii) history of hip‐related disease or diseases affecting the hip, such as infection, trauma, and neoplasm.

### *Participants*

We collected 135 3D CT images of bilateral views of the pelvis taken at a single hospital.

All CT scans were originally taken for examination of internal organs. We excluded 10 subjects because of the poor quality of the 3D CT data, and another 2 subjects due to lost 3D CT images. The images of the remaining 123 subjects, 64 men and 59 women, were analyzed by performing a virtual PAO (246 hips). We compared male and female pelvises. The mean (standard deviation [SD]) age of participants was 63.9 ± 13.9 years (male participants: 62.7 ± 13.6 years; female participants: 65.0 ± 14.7 years).

### *Operation Methods*

There were four steps or cutting planes for each virtual PAO. To attain standardized results, we defined all four cutting planes as precisely as possible based on the methods described in the published literature^6^, ^8^, ^9^.

#### *Osteotomy of the Anterior Portion of the Ischium*

**Figure 1:**
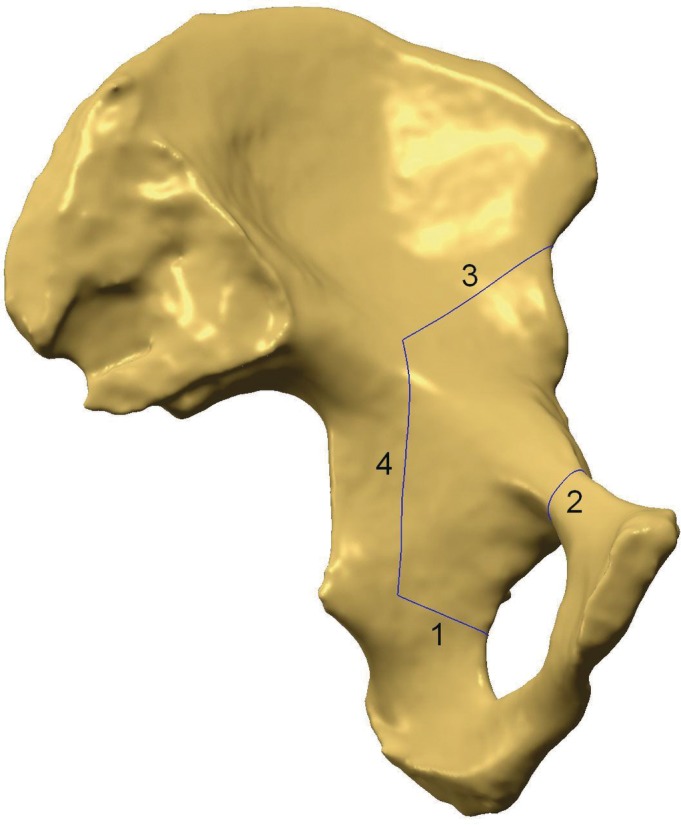
Medial pelvis view showing the periacetabular osteotomy cutting planes. 1, Virtual ischium osteotomy; 2, Virtual ramus superior os pubis osteotomy; 3, Virtual ilium osteotomy; 4, Virtual retroacetabular cutting.

**Figure 2:**
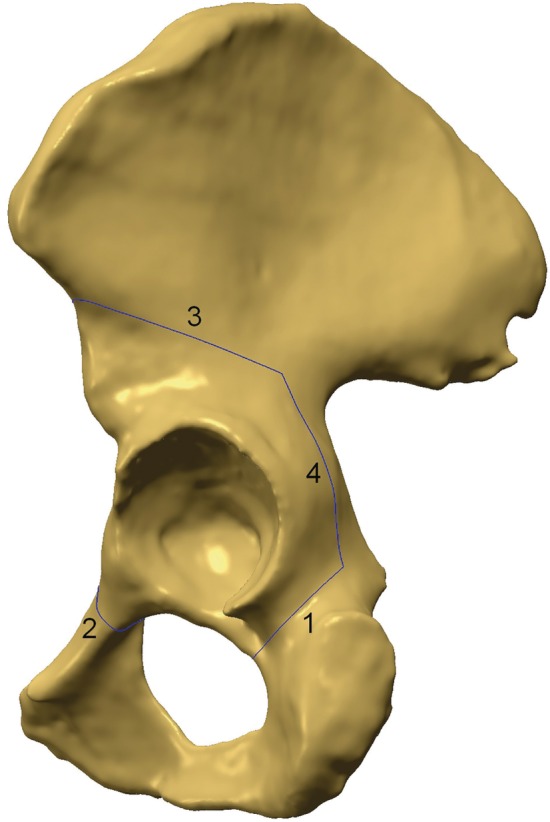
Lateral pelvis view showing periacetabular osteotomy cutting planes. 1, Virtual ischium osteotomy; 2, Virtual ramus superior os pubis osteotomy; 3, Virtual Ilium osteotomy; 4, Virtual retroacetabular cutting.

The first step was the osteotomy of the anterior portion of the ischium, and the first cutting plane was defined as “1 cm below the inferior lip of the acetabulum, along with the lowest curvature of the infracotyloid groove and toward or slightly above the ischial spine.” We call this the “ischial cutting plane” (Figs 1 and 2).

#### *Osteotomy of the Superior Pubic*

The next step was the osteotomy of the superior pubic ramus and its definition was “perpendicular to the long axis of the superior pubic ramus and 1 to 2 cm medial to the iliopectineal eminence.” We call it the “superior pubic ramus cutting plane” (Figs 1 and 2).

#### *Osteotomy of the Supraacetabular Ilium*

The osteotomy of the supraacetabular ilium was the third step and the third cutting plane was “just distal to the anterior superior iliac spine and toward the greater apex notch.” We call it the “iliac wing cutting plane” (Figs 1 and 2).

#### *Osteotomy of the Posterior Column*

**Figure 3:**
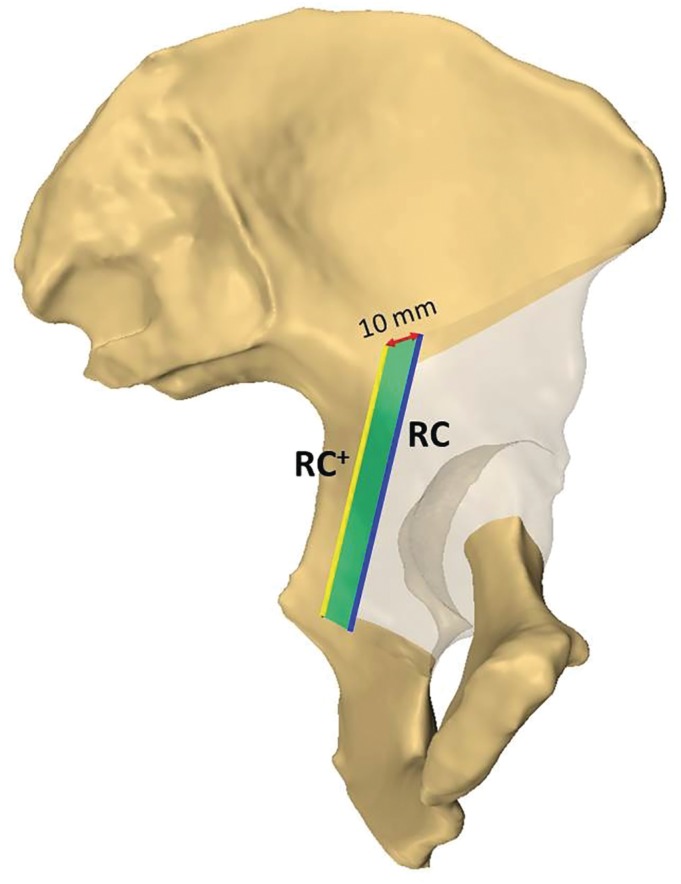
We provided two options for the retroacetabular cutting plane. The first one was the RC+ plane (depicted with yellow), which was defined as “passed through the crosspoint where the iliopectineal line and the iliac wing cutting plane meet.” The second option was the RC plane (depicted with blue), which was defined as “10 mm anterior to RC+ plane and parallel to it totally.” The 10‐mm shift is measured along the red line lying medially on the iliac wing cutting plane. The bone volume being the difference between these two options is depicted with green.

The last step was osteotomy of the posterior column and this cutting plane was “parallel to the edge of the sciatic notch and perpendicular to the axial plane of the posterior column”, and we call it the “retroacetabular cutting plane” (Figs 1, 2, 3). We provided two options for the retroacetabular cutting plane: the first one was defined as “passed through the crosspoint where the iliopectineal line and the iliac wing cutting plane meet” and was named the “RC^+^ plane+” (Fig. 3). The second option was defined as “10 mm anterior to the RC^+^ plane and parallel to it totally,” and was named the RC plane (see Fig. 3).

**Figure 4:**
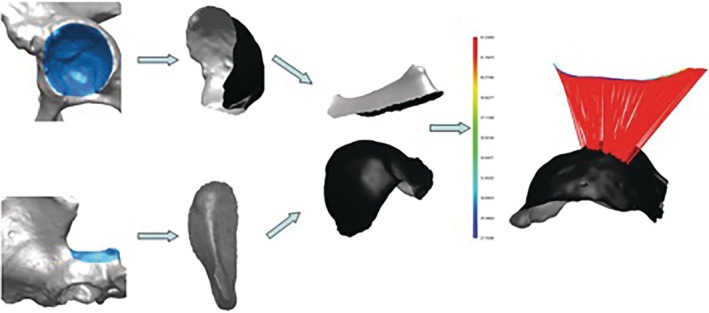
Measurement of the minimum distance between the acetabulum and the sciatic notch. It requires image processing operations on the pelvis surface segmented from the patient's CT data. It consists of the following steps (from left to right): delineation of the acetabulum and the greater sciatic notch region surfaces (marked in blue), separation of both regions from the rest of the virtual pelvis surface, determination of the greater sciatic notch curve, and measuring of the closest distance between the curve and the acetabulum surface. All these operations have to be performed for each CT dataset.

Three anatomical parameters were measured for each side: (i) the minimum distance between the acetabulum and the sciatic notch; (ii) the minimum distance between the acetabulum and the RC plane (osteotomy of the posterior column); and (iii) the length of the osteotomy line of the posterior column measured from the medial side of the pelvis (Fig. 4). For the latter two parameters, measurements were performed for both the RC^+^ plane and the RC plane. Subsequently, we calculated the difference between the minimum distance between the acetabulum of the RC plane and the RC^+^ plane, respectively. The mean value and the SD were calculated for each parameter.

Two software packages were used for image processing: the virtual Bernese osteotomy was performed with our own segmentation and surgery planning software ConeR (Version 1.0.0, Basel, Switzerland), and all the anatomical parameters were measured with the surface processing package Rapid Form 2006 (INUS Technology, Seoul, Korea)^10^.

### *Statistical Methods*

The results were analyzed statistically with SPSS 23.0 (IBM, Armonk, NY, USA) to determine the influence of gender differences on the results of the measurements. The results are shown as means with 95% confidence intervals (CI). A *t‐*test for independent samples and a χ^2^‐test were used to compare the means. A *P*‐value less than 0.01 (*P* < 0.01) was considered statistically significant.

## Results

### *Minimum Distance between the Acetabulum and the Sciatic Notch*

Among all patients, the mean (SD) minimum distance between the acetabulum and the sciatic notch was 25.82 ± 3.52 mm (95% CI, 25.36–26.25 mm; Table 1). The mean (SD) minimum distance was 27.18 ± 3.47 mm (95% CI, 26.56–27.78 mm) in men (Table 2), and 24.34 ± 2.92 mm (95% CI, 23.82–24.89 mm) in women (Table 2). The minimum distance between the acetabulum and the sciatic notch (Table 1) in men is on average 3.0 mm larger than in women (*P* < 0.001).

**Table 1:** Results of all patients in terms of different sides (n = 123)

Parameter	Subgroup	Overall (mm)		Left (mm)		Right (mm)		*P*‐value*
		Mean (SD)	95% CI	Mean (SD)	95% CI	Mean (SD)	95% CI	—
1	‐	25.82 (3.52)	25.36–26.25	25.81 (3.51)	25.19–26.44	25.83 (3.56)	25.20–26.47	0.895
2	RC^+^ plane	16.16 (3.69)	15.69–16.62	15.91 (3.68)	15.25–16.57	16.40 (3.69)	15.74–17.06	0.128
	RC plane	9.13 (3.83)	8.65–9.61	8.86 (3.78)	8.19–9.54	9.40 (3.86)	8.71–10.09	0.031
	*P*‐value†	<0.001	—	<0.001	—	<0.001	—	—
3	RC^+^ plane	60.89 (5.95)	60.14–61.63	61.21 (6.10)	60.12–62.29	60.57 (5.80)	59.53–61.61	0.051
	RC plane	67.67 (5.81)	66.94–68.4	68.09 (5.99)	67.02–69.16	67.24 (5.62)	66.24–68.25	0.036
	*P*‐value†	<0.001	—	<0.001	—	<0.001	—	—
4	‐	6.97 (0.65)	—	6.93 (1.07)	—	6.89 (1.12)	—	0.481

1, The minimum distance between the acetabulum and the sciatic notch

2, The minimum distance between the acetabulum and the retroacetabular cutting plane (osteotomy of the posterior column)

3, The length of the osteotomy line of the posterior column measured from the medial side of the pelvis

4, The difference of parameter‐2 (the minimum distance between the acetabulum and the retroacetabular cutting plane) between RC^+^ and RC plane

* Compared with left and right

† Compared with RC^+^ plane and RC plane in the same parameter; distance unit: mm.

**Table 2:** Results of all patients in terms of gender differences (mm)

Parameter	Subgroup	Male (n = 64)		Female (n = 59)		*P*‐value*
		Mean (SD)	95% CI	Mean (SD)	95% CI	
1	—	27.18 (3.47)	26.56–27.78	24.34 (2.92)	23.82–24.89	<0.001
2	RC^+^ plane	17.26 (3.67)	16.62–17.90	14.96 (3.33)	14.35–15.56	<0.001
	RC plane	10.23 (3.84)	9.55–10.90	7.94 (3.45)	7.31–8.57	<0.001
	*P*‐value†	<0.001	—	<0.001		—
3	RC^+^ plane	61.78 (6.75)	60.60–62.96	59.92 (4.77)	59.05–60.79	0.001
	RC plane	68.48 (6.65)	67.31–69.65	66.78 (4.60)	65.94–67.62	0.001
	*P*‐value†	<0.001	—	<0.001	—	—
4	—	7.04 (0.66)	—	7.02 (0.64)	—	0.728

1, the minimum distance between the acetabulum and the sciatic notch

2, the minimum distance between the acetabulum and the retroacetabular cutting plane (osteotomy of the posterior column)

3, the length of the osteotomy line of the posterior column measured from the medial side of the pelvis

4, the difference of parameter‐2 (the minimum distance between the acetabulum and the retroacetabular cutting plane) between RC+ plane and RC plane

* Compared with male and female

† Compared with RC^+^ plane and RC plane in the same parameter; distance unit: mm.

### *Minimum Distance between the Acetabulum and the Retroacetabular Cutting Plane (Osteotomy of the Posterior Column)*

The mean (SD) minimum distance between the acetabulum and the retroacetabular cutting plane (osteotomy of the posterior column) in the RC plane group was 9.13 ± 3.83 mm. The mean (SD) minimum distance was 8.86 ± 3.78 mm in the left hip group and 9.4 ± 3.86 mm in the right hip group (*P* = 0.031). The mean (SD) minimum distance was 10.23 ± 3.84 mm in men and 7.94 ± 3.45 mm in women (Table 1). The distance was on average 2.3 mm larger in men than in women (*P* < 0.001).

The mean (SD) minimum distance in the RC^+^ plane was 16.16 ± 3.69 mm overall, and 15.91 ± 3.68 mm for the left hip group and 16.40 ± 3.69 mm for the right hip group (*P* = 0.128). The mean (SD) minimum distance was 17.26 ± 3.67 mm in men and 14.96 ± 3.33 mm in women (Table 1); the minimum distance of male subjects was significantly larger than the minimum distance of female subjects (2.3 mm; *P* < 0.001).

We found that the minimum distance between the acetabulum and the retroacetabular plane for the RC^+^ plane was a mean of (SD) 6.97 ± 0.65 mm larger than for the RC plane (*P* < 0.001). This difference was also significant when considering the left and the right hip groups separately (Tables 2 and 3).

**Table 3:** Results of male patients (n = 64)

Parameter	Subgroup	Male overall (mm)		Left (mm)		Right (mm)		*P*‐value
		Mean (SD)	95% CI	Mean (SD)	95% CI	Mean (SD)	95% CI	
1	—	27.18 (3.47)	26.56–27.78	26.96 (4.08)	25.94–27.98	27.39 (2.79)	26.69–28.08	0.323
2	RC+ plane	17.26 (3.67)	16.62–17.90	16.94 (3.73)	16.01–17.87	17.59 (3.60)	16.69–18.49	0.178
	RC plane	10.23 (3.84)	9.55–10.90	9.93 (3.87)	8.96–10.90	10.52 (3.82)	9.57–11.48	0.110
	*P*‐value*	<0.001	—	<0.001	—	<0.001	—	—
3	RC^+^ plane	61.78 (6.75)	60.60–62.96	62.20 (7.08)	60.43–63.97	61.35 (6.44)	59.75–62.96	0.070
	RC plane	68.48 (6.65)	67.31–69.65	68.95 (6.95)	67.21–70.69	68.01 (6.36)	66.42–69.60	0.099
	*P*‐value*	<0.001	—	<0.001	—	<0.001	—	—
4	—	7.04 (0.66)	—	7.01 (1.08)	—	7.06 (1.10)	—	0.498

1, the minimum distance between the acetabulum and the sciatic notch

2, the minimum distance between the acetabulum and the retroacetabular cutting plane (osteotomy of the posterior column)

3, the length of the osteotomy line of the posterior column measured from the medial side of the pelvis

4, the difference of parameter‐2 (the minimum distance between the acetabulum and the retroacetabular cutting plane) between RC^+^ plane and RC plane

* Compared with RC^+^ plane and RC plane in the same parameter; distance unit: mm.

There was 1 female subject where the minimum distance between the acetabulum and the RC plane was 0 mm, which means that the osteotomy of the posterior column had perforated the hip joint.

### *Length of the Osteotomy Plane of the Posterior Column Measured from the Medial Side of the Pelvis*

The mean (SD) overall length of the osteotomy plane of the posterior column in the classical RC plane was 67.67 ± 5.81 mm. The mean (SD) length was 68.48 ± 6.65 mm in men, and 66.78 ± 4.60 mm in women (Table 1); the length was significantly (1.70 mm) shorter in the female group (*P* < 0.001).

The mean (SD) overall length in the RC^+^ plane was 60.89 ± 5.95 mm. The mean (SD) length was 61.78 ± 6.75 mm in men and 59.92 ± 4.77 mm in women (Table 1); the length was significantly (1.86 mm) shorter in the female group (*P* < 0.001).

We found that the mean (SD) length of the osteotomy in the RC^+^ plane was 61.78 ± 6.75 mm (approximately 7.0 mm) shorter than the mean (SD) length in the RC plane 68.48 ± 6.65 mm (*P* < 0.001). These differences were also shown in the left/right and male/female groups (*P* < 0.001). (Table 1, Table 3 and Table 4).

**Table 4:** Results of female patients (n = 59)

Parameter	Subgroup	Female overall (mm)		Left (mm)		Right (mm)		*P*‐value
		Mean (SD)	95% CI	Mean (SD)	95% CI	Mean (SD)	95% CI	—
1	—	24.34 (2.92)	23.82–24.89	24.57 (2.17)	24.00–25.14	24.15 (3.55)	23.22–25.07	0.262
2	RC^+^ plane	14.96 (3.33)	14.35–15.56	14.80 (3.31)	13.93–15.66	15.12 (3.36)	14.24–15.99	0.482
	RC plane	7.94 (3.45)	7.31–8.57	7.71 (3.36)	6.83–8.58	8.17 (3.55)	7.25–9.10	0.166
	*P*‐value*	<0.001	—	<0.001	—	<0.001	—	—
3	RC^+^ plane	59.92 (4.77)	59.05–60.79	60.12 (4.63)	58.92–61.33	59.72 (4.94)	58.43–61.00	0.369
	RC plane	66.78 (4.60)	65.94–67.62	67.15 (4.60)	65.95–68.35	66.41 (4.60)	65.21–67.61	0.188
	*P*‐value*	<0.001	—	<0.001	—	<0.001	—	—
4	—	7.02 (0.64)	—	7.09 (1.14)	—	6.94 (1.14)	—	0.141

1, the minimum distance between the acetabulum and sciatic notch

2, the minimum distance between the acetabulum and the retroacetabular cutting plane (osteotomy of the posterior column)

3, the length of the osteotomy line of the posterior column measured from the medial side of the pelvis

4, the difference of parameter‐2 (the minimum distance between the acetabulum and the retroacetabular cutting plane) between RC^+^ plane and RC plane

* Compared with RC^+^ plane and RC plane in the same parameter; distance unit: mm.

## Discussion

### *Complications in Periacetabular Osteotomy Surgery*

Periacetabular osteotomy surgery has been widely used for the treatment of hip diseases, such as developmental dysplasia in young adults, and has been associated with good outcomes. However, serious complications, including intra‐articular penetration, can occur^4^. There is a safe zone for the dorsal acetabulum cut during a periacetabular osteotomy that measures between 2 and 3.2 cm in either female or male European patients before the surgeon perforates either the sciatic notch or the acetabulum^4^. This widely used technique described by Ganz *et al.* could, however, also lead to complications, including intra‐articular penetration and sciatic nerve palsy after dorsal osteotomy^4^, ^7^. Other surgeons have developed different surgical techniques^11^, ^12^, ^13^, such as the direct anterior, the modified Smith–Peterson and the dual anterior–posterior approaches^14^ to overcome complications. However, Kim *et al.* 15 compared these techniques and found that there was no difference in patient outcomes, operative time or complication rates in single and double approaches. Therefore, the aim of our study was to provide useful anatomical data measurements to enable the surgeon to perform periacetabular osteotomy more safely, and to provide data on the optimal osteotomy distances based on 3D CT scans. Due to the complexity of the local anatomy, PAO remains a technically demanding procedure with a distinct learning curve for the orthopedic surgeon^5^, ^6^, ^7^, ^16^, ^17^. Although major morbidity only rarely occurs when the procedure is performed by skilled surgeons^17^, the overall complication rate is still approximately 20% to 25%, especially for relatively inexperienced surgeons.

#### *Anatomic Study in Periacetabular Osteotomy Surgery*

An anatomic study by Shiramizu *et al.* 18 assessed several anatomical measurements, and noted that the most important one was the minimum distance from the greater sciatic notch to the circular hole, which was similar to the minimum distance between the sciatic notch and the acetabulum. This mean (SD) distance was 15.4 ± 3.9 mm in their study. The authors speculated that the posterior margin of the hip was located approximately 2 cm anterior to the sciatic notch, and they did not find a statistical difference for any results between genders. This result is in line with our results of the mean (SD) minimum distance between the acetabulum and the retroacetabular cutting plane (osteotomy of the posterior column) in the new cutting plane of 16.16 ± 3.69 mm. However, our results indicate that there is a statistical difference between men 17.26 ± 3.67 mm and women 14.96 ± 3.33 mm, which may reflect gender‐based differences in anatomic structure.

#### *The Entry Point and Direction for Supraacetabular Osteotomy*

There are several descriptions of the entry point for supraacetabular osteotomy, but all go into the same direction (toward the sciatic notch) and have a similar ending point, which is 1 cm above the iliopectineal line (the iliac wing cutting plane). Ganz *et al.* 4 first described this entry point as “proximal to the anterior inferior iliac spine” in 1988. In 2001, Leunig *et al.* 9 moved the recommended entry point proximally, to the inferior border of the osteotomized anterior superior iliac spine. Clohisy *et al.* 19 introduced the most proximal iliac cut, which is made “just proximal to the osteotomy of the anterior superior iliac spine.” However, the following question remains: “Which entry point is optimal?” The answer is still unknown, but at present, the supraacetabular osteotomy line introduced by Leunig *et al.* 9 is used by most surgeons. In the present study, that point was the location of the iliac wing cutting plane. In the majority of the literature, the osteotomy of the posterior column (the retroacetabular cutting plane) is started from the same point (1 cm above the iliopectineal line), which we define as the RC plane.

The length of the osteotomy plane of the posterior column measured from the medial side of the pelvis shows that the osteotomy length in the RC^+^ plane is shorter than in the RC plane. Furthermore, the osteotomy length in female subjects was also shorter than in men. A shorter length of the osteotomy plane can reduce the risk of intra‐articular penetration as well as the difficulty of the operation. Similar results by Ferro *et al.* 8 also led to the recommendation. That retroacetabular cutting should keep a safe distance of 1 cm from the margin of the posterior column and should be parallel to it; complications were observed in 7 specimens (46%). In our research, the new RC^+^ plane may be nearly 10 mm away from the margin of the posterior column; therefore, the RC^+^ plane is safe for both the acetabulum and the margin of the posterior column. Nevertheless, a potential problem relating to the new RC^+^ plane is the strength of the remained column. The remaining continuity of the posterior column represents one of the advantages of PAO, permitting early mobilization after the surgery. Weakening of the posterior column strength may cause a breakage of continuity and inhibit early mobilization. In our research, only virtual osteotomies have been performed, and the numerical evaluation of the biomechanical strength of the resulted posterior column was beyond our computational resources.

#### *Gender Difference*

We found significant differences when comparing the data of female subjects with data of male subjects. The results for the minimum distance between the acetabulum and the retroacetabular cutting plane (osteotomy of the posterior column) and the minimum distance between the acetabulum and sciatic notch revealed much shorter distances in women than in men, which proves that the safety margin in female cases is narrower than in male cases. In addition, in 1 female case, the minimum distance between the acetabulum and the retroacetabular cutting plane was 0 mm, which means the osteotomy of the posterior column has penetrated the hip joint. Although we found no evidence that complications of periacetabular osteotomy have a direct relationship with the patient's gender, the present finding would suggest performing this procedure in female patients with caution.

#### *The Limitations and Advantages of This Study*

To the best of our knowledge, this is the first study that provides anatomic measurements from the pelvises of European patients, and that thereby clarifies the margin of the hip on a presumed quadrilateral surface.

The first limitation of this study is the virtual approach, and our data could not be confirmed by performing an actual PAO. This is particularly limiting concerning the caudal dorsal acetabular and ischiadic (sciatic) osteotomy that is curved, as we had to work with straight cutting planes. Second, the source patient selection was not representative for the age group of patients that usually undergo PAO, and stemmed from patients without real hip diseases. However, the anatomy of young adults and older patients changes more in the density of the bone than in the surface of the pelvic anatomy^20^. Third, although the safety space for PAO was 3 mm narrower in women than in men, this has no clinical implication, especially in a real operation. Further clinical research is needed to evaluate this narrower space in women.

Fourth, we only measured the distance between the bony structure, although vascular or neurologic injuries also represent common complications, especially injury of the sciatic nerve. We did not measure the distance between the sciatic nerve and the RC (RC^+^) plane but due to the inherently bone‐oriented CT imaging, it was difficult to make a precise measurement between bony structure and practically non‐detecteable soft tissue in the CT datasets used in our study. The pelvis data were segmented from abdominal CT scans which have a different quality compared to pelvis CT scans. Furthermore, there is a deviation of the tissue position between the CT scan and a real operation, especially the sciatic nerve moves with different intraoperative position^21^. We used millimeters as distance units, although it was hard to achieve in traditional visualized operation, when combined with preoperation CT screen, 3D printed guide plate, and navigator.

A personalized precise PAO may be possible in the near future. The advantages of this study include the large number of pelvises that were studied and the exact measurement method.

### *Conclusions*

In our study, the mean (SD) minimum distance between the acetabulum and the sciatic notch was 25.82 (3.52) mm. The safety space for PAO in women was narrower than in men. By shifting the RC plane 10 mm posteriorly, the RC+ plane can provide a safer cutting distance to avoid disastrous complications such as intraarticular penetration.
